# Letter I

**Published:** 1804-06-01

**Authors:** 


### LETTER I.

### Of Quacks and Empiricism.

WHEN prejudice is become general and sanctioned by time, to eradicate it is an Herculean task; but as your Journal has been the medium of diffusing science, and of appreciating medical fact, I am encouraged to hope that neither you nor your readers will deem a few pages misapplied, in the admission of some cursory remarks on empiricism; a subject which affords an ample revenue to the state, gives a bias for medicine in the community, and ultimately tends to promote the interests of the profession, by exciting and extending this propensity, and which rather increases than diminishes the number of patients of the regular practitioner, as quack medicines are generally calculated to amuse without curing the sick.

Imagination is the most active passion of the mind, at the same time it is one of the least stable; so that the persons most addicted to quackery are, such as are under the influence of more imaginary than real disease, the nervous, hypochondriac, and hysteric. They are objects, however, of compassion, and their sensibilities demand pity. They find solace in variety, and their minds are soothed and charmed by novelty; the benevolent will therefore be disposed to excuse and indulge them in a propensity so conducive to mental comfort. The greatest relief, indeed, is experienced by the medical professor, who is tired with importunity to remove imaginary evils, or to subscribe to unnecessary and unavailing recipes.

There is, in the human mind, a love for novelty and a predilection for the marvellous. It is predominant in moral and sometimes even in virtuous pursuits, hence the enthusiasm enthusiasm in religious, and the ardour in politics; the first to the excess of mania and suicide, and the latter to deliberate murder by duelling. It is not surprizing therefore that the love of the marvellous should extend to objects that respect health, when so many flattering promises (however fallacious) are held up to the desponding invalid, whose weakness leads him to credulity, and his distempered imagination to hope and confidence.

A circumstance highly conducive to quackery is, the overflowing wealth of this country. Persons of fortune sufficient to enable them to live without industry, (which is itself a disease, or the parent of disease) frequently fall into a state of ennui, which would become insupportable, were it not that in moral, as well as in physical evils, a disease when it shall have arisen to a certain extent, fortunately tends to cure itself; hence the privileged orders, those exempted from labour by their affluence, attempt to ward off the consequent evils, by cards, plays, parties, clubs, public breakfasts, public dinners, and nocturnal amusements; others, of a more hypochondriac temperament, enjoy the luxury of novels and patent medicines, and after satiety has been gratified by variety, occasionally repose upon regular practitioners, till the doctor, wearied out by importunity, and the patient by imaginary or real maladies, reverts to new nostrums, or by visiting fashionable watering places, dissipates care, and diverts ennui, at least for a season.

It is not my intention at present to convey the reader to times of yore; but I have lived to have known a distinguished group of quacks, whose names and remedies are now scarcely remembered; or if the latter are recollected, it is to mark the loss of their celebrity. Ward, about the middle of the last century, still lives in recollection, by the formulæ of medicines, bequeathed to a charitable purpose; whilst he was a prominent character, lived a Dr. Ryan, an obscure physician, of merit and abilities; but without professional character himself, he was the means of raising that of Ward, by giving or rather selling to him his nostrums, and by which Ryan must have raised a considerable sum, as he assured me he received two hundred pounds for his white drop alone. Soon afterwards Le Fevre from Liege appeared as a blazing star, and in a few months realized in this credulous country about ten thousand pounds, by pretending to cure the gout, a delusion he carried on by giving small doses of white vitriol. Although he was ignorant of medicine, he possessed the common common sense to know that the gout makes its attack principally in the spring and autumn, on which account he commenced his operation in the spring period, when the violence of the attack would be naturally receding, and he had the prudence to quit England (carrying home his fortune) before the autumnal return of the disease.

Some empirics have succeeded by the fascination of a title to a nostrum, thus the Ephractic Pill of the Dispensary, afforded a fortune to Dr H. by vending it under the name of the Female Pill, whence of course it must be inferred to be applicable to the case of every female. Daffy's elixir, was the old elixir salutis, now tincture of senna. Godbold's syrup is honey and vinegar, the composition of which was known before Godbold was born, as the simple oxymel of the shops.

In Surgery, quackery has been extended with equal profit to the empiric, although perhaps with more injury to the patient. A few years ago, Capt. *** came from America, with the patronage of numerous recommendation of being able radically to cure cancer; and although he never cured one, he is said to have pocketed ten thousand guineas of devoted English dupes.

This afforded encouragement to a more recent adventurer, a pupil of Dr. Rush, and really recommended by him; and whose reputation was so high in cancer empiricism, as to induce a lady here to offer him one thousand pounds to cross the Atlantic as a douceur. Weak minds are apt to estimate the value of an object by the difficulty of acquiring it; and the eclat of a single patient, although no advantage could be gained, except by the American adventurer, suddenly raised his reputation, and acquired him a fortune, which the sagacity of his countrymen never would have conferred.

After this general coup d'œil of the empirical field, I shall descend to particular characters, and introduce my second letter with some account of the celebrated Dr. Day, should you approve the first address of your constant reader.

IETROS.

*London, May* 20, 1804.

*Account*

